# Association of MTHFR rs1801133 and homocysteine with Legg–Calvé–Perthes disease in Mexican patients

**DOI:** 10.1186/s13023-022-02264-2

**Published:** 2022-03-09

**Authors:** José Guillermo Buendía-Pazarán, Edgar Hernández-Zamora, Armando O. Rodríguez-Olivas, Leonora Casas-Ávila, Margarita Valdés-Flores, Elba Reyes-Maldonado

**Affiliations:** 1 Morphology Department, Escuela Nacional de Ciencias Biológicas, Instituto Politécnico Nacional (ENCB, IPN), Prolongación de Carpio y Plan de Ayala s/n, Col. Santo Tomás, Miguel Hidalgo, C.P. 11340 Mexico City, Mexico; 2 Genomic Medicine, Instituto Nacional de Rehabilitación “Luis Guillermo Ibarra” (INR-LGII), Mexico City, Mexico

**Keywords:** Osteonecrosis, Thrombosis, LCPD, COL1A1, COL2A1, MTHFR, CBS, Prothrombin

## Abstract

**Background:**

Legg–Calvé–Perthes disease (LCPD) is the avascular osteonecrosis of the proximal femoral epiphysis. It is a rare disease of unclear etiology in children, although alterations in coagulation or the collagen gene have been described and could be associated with its etiology. Our objective was to evaluate the following alterations: COL1A1 (rs1107946, rs2412298), COL2A1 (rs121912891 and rs387106558), MTHFR rs1801133, CBS rs115742905, and PT rs1799963 and their relationship with LCPD.

**Methods:**

DNA was obtained and genotyped by real-time PCR with TaqMan probes. Prothrombin (FII) and homocysteine (Hcy) were determined by a coagulometric method. The variables were described as mean and standard deviation or percentages, and genotypic and allelic distributions were analyzed using the Student's t-test. The Hardy–Weinberg equilibrium and OR were also used.

**Results:**

We studied 23 patients with LCPD and 46 controls. We did not find any association of the MTHFR, CBS, PT, COL1A1, and COL2A1 genetic variants with LCPD. However, when adjusting the data with the Hcy values for the MTHFR C677T polymorphism, the C/C genotypes showed an association with the recessive model (*p* = 0.038), with susceptibility to LCPD.

**Conclusion:**

No association was found with the CBS, PT, COL1A1, and COL2A1 genes. Nevertheless, our results suggest a significant link between moderately elevated Hcy levels and the MTHFR C677T polymorphism in a cohort of Mexican children with LCPD.

## Introduction

Due to its low prevalence, Legg–Calvé–Perthes disease (LCPD) is rare, particularly in Latin America. However, the avascular osteonecrosis of the femoral head does occur in the pediatric population [1, 2]. The interruption of the blood supply causes fragmentation and deformation of the femoral head, which leads to mechanical hip alterations [3]. Although there are several proposed causes, its etiology is still unknown. Thrombophilia is one of the most widely accepted since the tendency to develop states of hypercoagulability could lead to the development of necrosis [4–6]. On the other hand, described collagen gene alterations could also be related to the etiology of the disease [7].

The methylenetetrahydrofolate reductase enzyme (MTHFR), together with the Cystathionine β-synthase (CBS), regulates the serum homocysteine (Hcy) level. MTHFR C677T and CBS T833C polymorphisms, Hcy levels, and other risk factors like polymorphisms of prothrombin G20210A (PT G20210A) have been linked to the development of prothrombotic states [8]. Various studies have suggested that these conditions could be related to the etiology of LCPD [9, 10].

LCPD consists of sequential stages of involvement of the proximal femoral epiphyses, including subchondral fracture, fragmentation, reossification, and healing with residual deformity. Even though genetic factors have been linked to the etiology of LCPD, the causal gene has not been identified. Nonetheless, COL2A1 gene mutations have been implicated in the etiology of LCPD because collagen is the essential matrix protein of all connective tissues. Some of these alterations could cause structural defects of type II collagen related to the major clinical aspects [11, 12].

On the other hand, the COL1A1 gene encodes the alpha-1 protein chain of type I collagen, the primary protein of bones. Some research focused on the COL1A1 polymorphisms associates them significantly with low bone mineral density, osteoporosis, increased fracture risk, and osteonecrosis [13, 14]. Also, the harmful effect of Hcy on bones may be mainly mediated by the binding of the molecule to collagen, which interferes with the formation of type I collagen cross-links [15]. Furthermore, several studies report a significant relationship between hypercoagulability, elevated levels of Hcy, and osteonecrosis [16, 17]. Because of the above, our objective is to evaluate the following alterations involved with coagulation genes, which could be associated with LCPD etiology: MTHFR C677T rs1801133, CBS T833C rs115742905, and PT G20210A rs1799963; the following collagen-related polymorphisms: COL1A1 G1997T rs1107946, COL1A1 C1663T rs2412298, COL2A1 G2306A rs121912891, and COL2A1 G3665A rs387106558; and their relationship with Mexican patients suffering from LCPD.

## Methods

We carried out a case–control study. The cases were first-time or recurrent patients of all ages, of both sexes, with a clinical and radiological diagnosis of LCPD, without other bone diseases or diseases related to coagulation abnormalities, and without any pharmacological treatment. Additionally, we matched healthy controls with the patients in a 2:1 proportion by age and sex, with radiological studies showing that the controls did not have any alterations in the femur or hip, had no history of hematological or thrombotic pathologies, and were not under pharmacological treatment.

All patients were recruited from the Orthopedic Service at Instituto Nacional de Rehabilitación Luis Guillermo Ibarra Ibarra (INR-LGII) in Mexico City. In addition, we obtained weight (kg), height (m), and body mass index (BMI).

### Genotyping

We obtained DNA from peripheral blood leukocytes. Genotyping was performed by real-time PCR with TaqMan probes in the conditions recommended by the manufacturer (Applied Biosystems, Foster City, CA). Briefly, reactions were performed in 25 ml volume, containing TaqMan PCR master mix (1X), TaqMan probe (100 nm), primers (900 nm of each), and DNA (25 ng). Samples were run in a StepOne Real-Time PCR System (Applied Biosystems). Cycling conditions: one cycle at 95 °C for 10 min, followed by 40 cycles at 95 °C for 15 s, and a final cycle at 60 °C for 1 min.

### Determination of coagulation factor II (prothrombin) and Hcy

Plasma was obtained from peripheral blood in tubes with 3.8% sodium citrate. The hemosiLTM 0020012200 chromogenic kit and the HemosiLTM 0020007800 kit for Hcy were used in ACL-7000 elite pro equipment to determine the prothrombin or coagulation factor II (FII). The results were expressed as a percentage of activity, reference range: 98 to 136% for FII; and in µmol/L, reference range: 10 to 14.4 for µmol/L Hcy.

### Statistical analysis

The variables are described as mean ± standard deviation or percentages (%) where appropriate. Differences in genotypic and allelic distribution between case and control groups were analyzed using Fisher's exact test or Student's t-test. The Hardy–Weinberg equilibrium (HWE) was calculated for the genotype frequencies in cases and controls using a Chi-square goodness of fit test in SNPStats. The odds ratio (OR) and the confidence interval (95% CI) were calculated to assess the relative risk of having the disease according to the relative frequency of different genotypes between cases and controls with PAST 4.03. Furthermore, the inheritance patterns for SNPs and associations were estimated using SNPStats (http://www.snpstats.net/start.htm).

### Ethical aspects

The cases were patients with LCPD. Controls were selected under the official Mexican standard NOM-253-SSA1-2012 guidelines, all participants received oral and written information, and parents or guardians signed a letter of consent. This study was approved by the INR-LGII research and ethics committees.

## Results

This study included 69 subjects: 23 patients (21 men and 2 women) of 3 to 18 years of age and 46 controls (42 men and 4 women). The age of all participants ranged from 3 to 18 years, with a mean (X) of 16.8 and a standard deviation (SD) of ± 11.6 (patients X = 16.8 ± 11.3, and controls X = 16.3 ± 11.0). The average height in patients was 1.48 ± 0.23 m compared to 1.49 ± 0.23 m in controls. The average weight was 46.21 ± 15.02 kg in patients, compared to 53.83 ± 24.06 kg in controls, without significant difference between patients and controls. Similarly, BMI presented no significant differences between patients and controls.

**Fig. 1:**
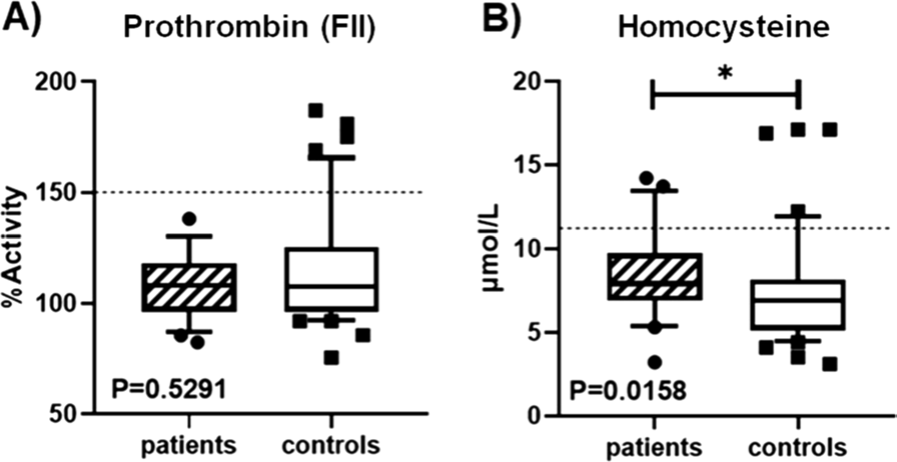
Comparison of **A** Factor II (prothrombin) and **B** Homocysteine. Mustache graphs showing the median and the 10–90 percentile. Out-of-range values (•, • •, ■, ■■, outliers) are represented with dots outside the mentioned percentiles. The *p* value from the comparison between patients and controls is also shown. Compared samples that showed a significant difference according to the Mann–Whitney U-test are highlighted with an asterisk (*), dotted line = maximum proposed reference value for children (····)

Prothrombin activity did not significantly differ between the patients' mean and the controls. However, regarding the levels of Hcy, there were substantial differences in the mean of the patients compared to the controls (Fig. 1).

The genotype frequencies of the MTHFR rs1801133 polymorphism were in Hardy–Weinberg equilibrium (HWE) because the mutant allele is present in controls and patients (*p* = 0.76 and *p* = 0.40, respectively). The CBS rs115742905 and PT rs1799963 variants were not found in HWE because the mutant allele is absent in either case. The wild genotype resulted in 100% in both populations (Table 1). The approach to the disease is complicated because its etiology is not yet clear.

**Table 1:** Association of MTHFR rs1801133, CBS rs115742905, PT rs1799963, COL1A1 rs1107946 and rs2412298, COL2A1 rs121912891 and rs387906558 with Legg–Calvé–Perthes Disease

SNP and model	Genotype	Controls n (%)	Cases n (%)	OR (95% CI)	*p* value^**#**^	OR (95% CI)*	*p* value*
MTHFR rs1801133							
	T/T	15 (32.6%)	6 (26.1%)	1.00		1.00	
Codominant	C/T	21 (45.6%)	9 (39.1%)	1.07 (0.31–3.66)		0.43 (0.08–2.30)	
	C/C	10 (21.7%)	8 (34.8%)	2.00 (0.53–7.54)	0.51	3.69 (0.56–24.56)	0.07
Dominant	T/T	15 (32.6%)	6 (26.1%)	1.00		1.00	
	C/T-C/C	31 (67.4%)	17 (73.9%)	1.37 (0.45–4.19)	0.58	0.97 (0.23–3.99)	0.96
Recessive	T/T-C/T	36 (78.3%)	15 (65.2%)	1.00		1.00	
	C/C	10 (21.7%)	8 (34.8%)	1.92 (0.63–5.81)	0.25	5.62 (1.02–30.93)	**0.038**
CBS rs115742905							
	T/T	46 (100.0%)	23 (100.0%)	Monomorphic SNP		Monomorphic SNP	
	T/C	0 (0.0%)	0 (0.0%)				
	C/C	0 (0.0%)	0 (0.0%)				
PT rs1799963							
	G/G	46 (100.0%)	23 (100.0%)				
	G/A	0 (0.0%)	0 (0.0%)	Monomorphic SNP		Monomorphic SNP	
	A/A	0 (0.0%)	0 (0.0%)				
COL1A1 rs1107946							
	G/G	21 (45.6%)	13 (56.5%)	1.00		1.00	
Codominant	G/T	13 (28.3%)	6 (26.1%)	0.75 (0.23–2.45)	0.63	0.73 (0.22–2.42)	
	T/T	12 ((26.1%)	4 (17.4%)	0.54 (0.14–2.03)		0.53 (0.14–2.00)	0.62
Dominant	G/G	21 (45.6%)	13 (56.51%)	1.00		1.00	
	G/T-T/T	25 (54.4%)	10 (43.5%)	0.60 (0.17–2.11)	0.41	0.63 (0.23–1.75)	0.38
Recessive	G/G-G/T	34 (73.9%)	19 (82.6%)	1.00		1.00	
	T/T	12 (26.1%)	4 (17.4%)	0.60 (0.17–2.11)	0.41	0.59 (0.17–2.10)	0.41
COL1A1 rs2412298							
	C/C	39 (84.8%)	19 (82.6%)	1.00		1.00	
	C/T	7 (15.2%)	4 (17.4%)	1.17 (0.31–4.50)	0.82	1.18 (0.31–4.55)	0.81
COL2A1 rs121912891							
	G/G	46 (100.0%)	23 (100.0%)	Monomorphic SNP		Monomorphic SNP	
	G/A	0 (0.0%)	0 (0.0%)				
	A/A	0 (0.0%)	0 (0.0%)				
COL2A1 rs387906558							
	G/G	46 (100.0%)	23 (100.0%)	Monomorphic SNP		Monomorphic SNP	
	G/A	0 (0.0%)	0 (0.0%)				
	A/A	0 (0.0%)	0 (0.0%)				

Significant values are shown in bold

*Adjusted by age

^#^Raw data

The genotype frequency of the COL1A1 rs1107946 variant was not found in HWE with respect to the controls. No significant differences were observed for the genotype and allelic distribution for the polymorphism between the patients with LCPD and the controls. The COL1A1 variant rs24122948 was found in HWE, but no differences were observed. In the case of COL2A1 rs24122948 and rs106558, we did not find the presence of the mutant alleles (Table 1).

Table 1 presents the link of MTHFR to age, which showed a significant association with the recessive inheritance model and was detected among carriers of the MTHFR CC compared to carriers of MTHFR TT + CT, regardless of age. However, apparently, a worse outcome is more closely related to an older age.

## Discussion

The prevalence of LCPD is highly variable in different populations. Since it is a rare disease, few reports in Mexico and Latin America describe it [18–20]. Children with LCPD begin showing pain in one or both legs and a slight limp, sometimes attributed to falls. On occasion, the pain goes away temporarily, but the lameness continues. Generally, the disease encompasses a broad spectrum of symptoms, from mild with no long-term sequelae to severe with a permanent degenerative change of the hip joint. Obtaining a timely and accurate diagnosis for a rare disease like LCPD can often be challenging due to the lack of experienced healthcare providers. In addition, research activities are less common, and, in developing countries, the problems are compounded by other resource limitations [21, 22]. Therefore, the correct diagnosis is only given when patients finally go to specialized pediatric orthopedic institutions, such as the INR-LGII.

The approach to the disease is complicated because its etiology is not yet clear. However, the main priority is to make an early diagnosis and offer a conservative or surgical treatment that keeps the head of the femur and the hip joint in the best conditions. For this reason, it is vital that primary care physicians know and understand this disease.

Genes involved in bone remodeling and architecture have been studied, and elevated levels of Hcy have been linked to osteonecrosis [17]. Because of this, we decided to explore variants in the COL1A1 gene that have been studied in relation to osteoporosis and fractures [23, 24]. However, none of these variants was informative in the studied sample of Mexican patients with LCPD. It has also been suggested that COL2A1, involved in osteonecrosis of the femoral head, is related to skeletal dysplasia due to failed cartilage development and growth [25]. However, none of these alterations had significant differences between the patients and the controls studied. Additionally, COL2A1 is suggested to be one of the representative causal genes, as Li et al. found a mutation in COL2A1 G1888A in a Chinese family affected by LCPD and osteonecrosis of the femoral head [12, 26], and Miyamoto et al. identified a mutation in COL2A1 G3508A in a Japanese family with LCPD. However, these findings differ from our results since we found no relationship with LCPD [27].

The PT G20210A mutation increases the risk of thrombosis [28]. López et al. studied 90 children with LCPD, and no patient had a family or personal history of early thrombotic events. However, compared to controls, four children with LCPD (4.4%) were heterozygous for the G20210A polymorphism but without significant association [29]. Vosmaer et al. found an increase in the incidence of LCPD in the presence of the prothrombin mutation when the FVL polymorphism was also present [30]. Our study did not find significant differences that relate this polymorphism, PT rs1799963, with LCPD.

Common alterations in some genes related to Hcy metabolism, such as MTHFR C677T and CBS T833C, have been shown to cause increased plasma homocysteine levels, thus bringing about a predisposition to thrombosis [31]. Our study did not find these variants, CBS T833C rs5742905, neither in controls nor in patients. Azarpira et al. showed that the MTHFR C677T polymorphism was not associated with the risk of LCPD in Iranian children [32]. When we analyzed the results for the MTHFR C677T rs1801133 polymorphism, they did not present a significant association with the risk of susceptibility to LCPD. However, the mutant allele frequency was higher than in the Iranian population, even though several epidemiological case–control studies have found that MTHFR polymorphisms could not play a significant role in the susceptibility to the development of osteonecrosis on the femoral head [33, 34]. In this study, we also found that the levels of Hcy between cases and controls present a significant difference (*p* < 0.05). In addition, when adjusting the value of the MTHFR C677T polymorphism with age, we found a significant association in the recessive model (OR 5.62 (1.02–30.93), *p* = 0.038). Age is one of the main factors that can influence a poor prognosis for patients.

In general, hyperhomocysteinemia is a risk factor for various diseases: vascular, neurological, diabetes, psoriasis, cancers, osteoporosis, among others [35]. In addition, Hcy is thought to alter bone remodeling [36]. Furthermore, it has been described that short-term moderate hyperhomocysteinemia affects bone and cartilage characteristics. Trabecular bone microarchitecture is especially sensitive to hyperhomocysteinemia. It shows clearly negative bone balance, mainly due to a decrease in trabecular numbers and markedly reduced trabecular connections, which indicates multiple alterations in collagen due to homocysteine accumulation in bones, indicative of broken collagenous cross-links [37].

One of the weaknesses of this work was that it required considerable time to capture this small population of patients, which poses difficulties in research. However, the results strengthen and support the theories that describe thrombosis and homocysteine as risk factors in developing LCPD. In addition, it contributes to the scientific knowledge and information about the disease in the Mexican population.

## Conclusions

This is the first study to evaluate genetic aspects of coagulation mediators and type I and II collagen in Mexican patients with LCPD. We did not find a relationship of collagen alterations (COL1A1 rs1107946, COL1A1 rs2412298, COL2A1 rs121912891, and COL2A1 rs387106558) or coagulation alterations (CBS T833C rs115742905 and PT G20210A rs1799963) with LCPD. However, we found an association of the MTHFR rs1801133 polymorphism in a recessive model adjusted by age and elevated levels of Hcy in a population of Mexican children with LCPD.

## Data Availability

All relevant data used in this study are included in the manuscript. The corresponding author can be contacted if any further information is needed.

## Abbreviations

LCPD: Legg–Calvé–Perthes disease
COL1A1: Collagen type I alpha 1 chain
COL2A1: Collagen type II alpha 1 chain
MTHFR: Methylenetetrahydrofolate reductase
CBS: Cystathionine beta-synthase
PT: Prothrombin
DNA: Deoxyribonucleic acid
Hcy: Homocysteine
INR-LGII: Instituto Nacional de Rehabilitación “Luis Guillermo Ibarra Ibarra”
BMI: Body mass index
FII: Coagulation factor II
HWE: Hardy–Weinberg equilibrium
OR: Odds ratio
CI: Confidence interval
SNPs: Single nucleotide polymorphisms
NOM: Norma Oficial Mexicana
